# Disease Combinations Associated with Physical Activity Identified: The SMILE Cohort Study

**DOI:** 10.1155/2016/9053578

**Published:** 2016-01-04

**Authors:** Sarah Dörenkamp, Ilse Mesters, Jan Schepers, Rein Vos, Marjan van den Akker, Joep Teijink, Rob de Bie

**Affiliations:** ^1^Department of Epidemiology and CAPHRI School for Public Health and Primary Care, Functioning and Rehabilitation Programme, Maastricht University, 6229 ER Maastricht, Netherlands; ^2^Department of Methodology and Statistics, CAPHRI School for Public Health and Primary Care, Maastricht University, 6229 ER Maastricht, Netherlands; ^3^Department of Medical Informatics, Erasmus University Rotterdam, 3015 CE Rotterdam, Netherlands; ^4^Department of Family Medicine, CAPHRI School for Public Health and Primary Care, Maastricht University, 6229 ER Maastricht, Netherlands; ^5^Department of General Practice, Catholic University of Leuven, 3000 Leuven, Belgium; ^6^Department of Vascular Surgery, Catharina Hospital, 5623 EJ Eindhoven, Netherlands

## Abstract

In the search of predictors of inadequate physical activity, an investigation was conducted into the association between multimorbidity and physical activity (PA). So far the sum of diseases used as a measure of multimorbidity reveals an inverse association. How specific combinations of chronic diseases are associated with PA remains unclear. The objective of this study is to identify clusters of multimorbidity that are associated with PA. Cross-sectional data of 3,386 patients from the 2003 wave of the Dutch cohort study SMILE were used. Ward's agglomerative hierarchical clustering was executed to establish multimorbidity clusters. Chi-square statistics were used to assess the association between clusters of chronic diseases and PA, measured in compliance with the Dutch PA guideline. The highest rate of PA guideline compliance was found in patients the majority of whom suffer from liver disease, back problems, rheumatoid arthritis, osteoarthritis, and inflammatory joint disease (62.4%). The lowest rate of PA guideline compliance was reported in patients with heart disease, respiratory disease, and diabetes mellitus (55.8%). Within the group of people with multimorbidity, those suffering from heart disease, respiratory disease, and/or diabetes mellitus may constitute a priority population as PA has proven to be effective in the prevention and cure of all three disorders.

## 1. Introduction

Multimorbidity, defined as the coexistence of two or more chronic diseases, is progressively more prevalent with age [1–3]. Patients with multimorbidity tend to have a poorer functional status, diminished quality of life and make more use of ambulatory and inpatient healthcare [4]. However, the growing prevalence of patients with multiple chronic diseases not only is the result of ageing and advances in medical care, but is also related to modifiable factors like unhealthy lifestyle behaviours; various studies have shown a strong association between an unfavourable lifestyle and many chronic diseases [5–7]. It is therefore important to consider lifestyle as a relevant strategy for the secondary prevention and cure of multimorbidity in patients.

Regular physical activity (PA) has proven to be effective in the prevention and cure of chronic conditions [8]. An inverse relationship has been shown between regular PA and cardiovascular disease, thromboembolic stroke, hypertension, osteoporosis, diabetes mellitus type II, obesity, colon cancer, breast cancer, anxiety, and depression [9]. In a study of Kaplan et al. [10] the absence of thirteen chronic diseases was related to frequent PA. In addition to the association between PA and isolated chronic diseases, the association between PA and multimorbidity has recently been explored in older patients in a cross-sectional study by Autenrieth et al. [11]. This study showed an inverse relationship between PA and multimorbidity among men aged 65–94 years. We wish to stress here that the analysis of the study of Kaplan et al. [10] was based on the sum of 13 chronic diseases, while Autenrieth et al. [11] defined multimorbidity as the presence of ≥2 chronic diseases from a list of 13 diseases. Both studies used the sum of diseases as a measure for multimorbidity. Using the summation of diseases as a measure of multimorbidity has been criticised as comparing apples and oranges [12]. The resulting composite expresses multimorbidity in an additive form. A more comprehensive approach is suggested that takes into account how chronic diseases are distributed and aggregate in the population, whereby any clustering of chronic diseases keeps the unique contribution of each disease salient [13]. In addition, it allows an examination of how specific combinations of chronic diseases may interact to affect physical activity behaviour. We hypothesise that certain combinations of chronic diseases may present a stronger association with physical activity as previous studies have already shown that the cumulative effect of chronic diseases is not simply additive [12]. Awareness of the association between specific combinations of chronic diseases and limited physical activity levels could facilitate the development of more targeted counselling strategies and treatment plans.

Prior work has shown an inverse relationship between the number of chronic diseases and physical activity. Yet, to our knowledge no study has assessed the association between specific disease clusters and physical activity. This study therefore goes beyond prior work in the field of multimorbidity and investigates which clusters of multiple chronic diseases are associated with PA in a large representative sample of older Dutch people above 55 years of age, measured in compliance with the Dutch PA guideline.

## 2. Method

### 2.1. Study Design and Setting

This cross-sectional study is part of a dynamic prospective cohort study, the Study of Medical Information and Lifestyles in Eindhoven (SMILE), the Netherlands. The SMILE cohort study was performed between 2002 and 2010 and was a joint project between Maastricht University and the Eindhoven Corporation of Primary Health Care Centres (SGE), including nine centres representing 32 general practitioners. Data for the SMILE cohort study was collected in two ways: (1) information on morbidity, mortality, medication use, and healthcare facility utilisation was continuously registered using electronic medical records (EMRs) in the nine primary healthcare centres and (2) information on lifestyles and chronic diseases was collected by using annual self-administered paper questionnaires. Information on physical activity was collected annually in November. The self-reported chronic disease questionnaire was collected annually in May among all adults aged 55 years and older. The SMILE study protocol has been published [14] and approved by the Medical Ethics Committee of the Maastricht Academic Hospital (MEC 07-4-030). To enhance transparency and reproducibility, this paper has been written according to the STROBE checklist for cohort studies.

### 2.2. Participants

Registrees (12 years and older) of the participating healthcare centres were invited to participate in the overall study. All patients signed informed consent forms. Adult data (from patients aged 55 years and older) from 2003 was used in the present study since that year included the largest number of patients who completed both questionnaires (*n* = 3,386).

### 2.3. Variables

Compliance with the Dutch PA guideline, which states that every adult should accumulate 30 minutes or more of moderate intense physical activity (4 METs) on at least five, or preferably all, days of the week [15], was the primary outcome measurement (1 = compliance with the guideline; 0 = no compliance with the guideline). Cluster variables included the presence or absence of 15 self-reported chronic diseases. The derived clusters operated as independent variables.

### 2.4. Data Sources/Measurement

Data about the level of physical activity came from the adult questionnaire and self-reported chronic diseases data was extracted from the 55+ questionnaire.

#### 2.4.1. Short Questionnaire to Assess Health-Enhancing Physical Activity (SQUASH)

Physical activity was measured by the “Short Questionnaire to Assess Health-Enhancing Physical Activity (SQUASH)” [15]. Patients were asked to refer to an average week in the past few months. The SQUASH questionnaire was structured in a way that made it possible to assess compliance with the Dutch PA guideline. The SQUASH consists of three main queries: number of active days per week, average time per day, and intensity. All physical activities were prestructured in (a) commuting activities, (b) leisure-time activities, (c) household activities, and (d) activities at work and at school. Examples for each category of physical activity (a–d) were given as activities at work, household activities, and sports. Example activities were chosen based on an intensity of 4 METs but did not include light activities at work and light household activities. These light activities entail a considerable amount of time per day and therefore contribute to the habitual activity level. Moreover, in conformity with the SQUASH questionnaire, manual hobbies were excluded in the SQUASH due to their low MET values (~2 METs); however, hobbies that do have meaningful MET values were noted under sports.

An intensity score and a total activity score were allocated to all activities. Each activity was assigned a MET value using the Ainsworth compendium for physical activities, in which one MET is defined as the energy expenditure for sitting quietly [16]. For each intensity category, cut-off points were defined based on the Dutch PA guideline [15]. Activities between 1.6 and 2.9 METs were classified as lightly intense, between 3 and 5.9 METs as moderately intense, and ≥6 METs as vigorously intense [15, 16]. The total minutes of each activity were calculated by multiplying frequency (days/week) by duration (minutes/day).

#### 2.4.2. Self-Reported Chronic Disease Questionnaire

The presence or absence of 15 chronic diseases was measured using the self-reported chronic disease questionnaire. This questionnaire is based on a medical screening questionnaire of the Dutch Association of General Practitioners (LHV) [17]. Patients had to record their actual health status for the following fifteen chronic diseases: chronic bronchitis, emphysema, and asthma; heart disease or myocardial infarction; severe bowel disease; liver disease or cirrhosis; severe kidney disease; diabetes mellitus; malignancy or cancer; epilepsy; migraine; stroke or stroke-related complaints; inflammatory joint disease; rheumatoid arthritis; osteoarthritis of knees, hips, and hands; severe back problems, hernia, sciatica, or osteoarthritis; and persistent injury from an accident at home, in sports, school/work, or traffic. Data on chronic diseases were binary (1 = a given disease is present; 0 = a given disease is absent). An open question in the questionnaire allowed patients to add other present chronic diseases that were not listed in the questionnaire. To maximise the use of available data, all chronic diseases noted in the open question (*N* = 1,077) were incorporated in the data gathered from the completed self-reported chronic disease questionnaires. Two researchers, assisted by a medical specialist, separately assigned the diseases noted in the open question to the existing categories in the chronic disease questionnaire (JT).

### 2.5. Bias

Cluster analysis algorithms assume that there are no missing values. Solutions are developed if values are missing; however, these are only technically valid if the values are missing completely at random (MCAR). In the self-reported chronic disease questionnaires, missing values are observed ranging from 592 (17.5%) for epilepsy to 818 (24.2%) for inflammatory joint disease. We assume that these missing values are not completely random (MNAR) [18] but are the result of inadequate instructions being provided with the chronic disease questionnaire. Patients were asked to indicate in a dichotomous prestructured form (yes/no) which of the 15 chronic diseases they suffer from. The hypothesis is that a proportion of patients followed this instruction by only indicating the presence of a certain disorder without explicitly registering the absence (by ticking “no”) of all other diseases listed. Following this hypothesis, all missing values for the 15 chronic diseases were interpreted and recoded as “disease being absent.”

### 2.6. Statistical Analysis

The aim of the analysis was to identify clusters of chronic diseases based on their relative similarity or dissimilarity (distance). Cluster analysis is used because it best fits the aim of our study, namely, to identify meaningful groups of patients with chronic diseases. Because there is not a one-and-only valid approach to establish groups of patients in relation to chronic diseases, the two most frequently applied forms of clustering, namely, Ward's agglomerative hierarchical clustering and *K*-means clustering, were used.

First, the most widely used form of clustering [18–20], Ward's agglomerative hierarchical clustering, applying squared Euclidean distance as a similarity measure, was performed. Each individual disease starts as an individual cluster which is then gradually agglomerated with the next most similar cluster on the basis of a proximity measurement using a predefined fusion algorithm [19]. Distances are recalculated and diseases reassigned until all are in a single cluster. Robust groups of chronic diseases are obtained at the point where the individual clusters are as homogeneous as possible within clusters and as heterogeneous as possible in relation to the other clusters [20]. As the number of clusters was not known a priori, a series of cluster analyses with predefined cluster numbers ranging from 2 to 5 was performed. The agglomerative coefficient, the dendrogram, and the pseudo-*F* statistic were used to determine the appropriate number of clusters. The pseudo-*F* statistic (ratio of the mean sum of squares between groups to the mean sum of squares within groups [20]) was calculated to capture the “tightness” of clusters. The following formula was used to calculate the pseudo-*F* statistic:

(1) $$\begin{array}{c} \text{Pseudo-}F=\frac{\left(SS\left(T\right)-SS\left(W\right)\right)/\left(N-1\right)}{WGSS/\left(n-N\right)}. \end{array}$$

In the above formula, SS(T) is the total sum of squares, SS(W) is the within-group sum of squares, and *N* is the number of clusters. A larger pseudo-*F* statistic indicates a better cluster solution. Second, based on the findings obtained from using Ward's agglomerative hierarchical clustering, a *K*-means cluster analysis was executed to check our findings. Unlike the hierarchical clustering method, *K*-means starts by assigning patients randomly to one cluster and proceeds with iteration. Patients were gradually reassigned to minimise the within-cluster variation. This iteration was continued until the smallest within-cluster variation was reached. One thousand combinations of random starts were investigated. Cross-tabulation using chi-square statistics was performed to assess the association between established clusters of chronic diseases and compliance with the Dutch PA guideline. To get full insight into the association between multimorbidity and physical activity and to study the consequences of branching of clusters Ward's two-to-five-cluster solution will be studied. Disease frequency distributions within each cluster were evaluated using crosstabs. The sociodemographic characteristics of all patients belonging to each cluster in each cluster solution were determined using descriptive and frequency statistics.

## 3. Results

### 3.1. Participants

Both the general adult questionnaire and the additional 55+ questionnaire were returned by 3,386 patients.

Fifty-three per cent were female and the average age of patients was 68 years (range: 55–95 years). The average length and bodyweight of patients were 1.70 m (range: 1.41–1.99 m) and 75 kg (range: 40 kg–185 kg), respectively. Osteoarthritis of knees, hips, and hands was the most prevalent disease (23%). The prevalence of heart disease or myocardial infarction was approximately twice as high in males as in females (11.3% versus 6.6%, resp.). In comparison, musculoskeletal disorders like inflammatory joint disease (7.2% versus 10.4%), rheumatoid arthritis (2.7% versus 6.0%), and osteoarthritis of knees, hips, and hands (18.2% versus 27.4%) were less prevalent among females compared with males (Table 1). Prevalence rates of all fifteen chronic diseases from the SMILE cohort (measured in the Eindhoven region) were comparable with national prevalence rates in Dutch older adults [21, 22].

### 3.2. Multimorbidity Clusters

*Two-Cluster Solution*. For Ward's agglomerative hierarchical clustering, the stepwise agglomerative coefficients and the pseudo-*F* statistic suggested a two-cluster solution being most feasible (Table 2). *K*-means clustering displayed consistent results, with the sum of squares (SS) being 2177.8 and pseudo-*F* being 1318.4.

**Figure 1:**
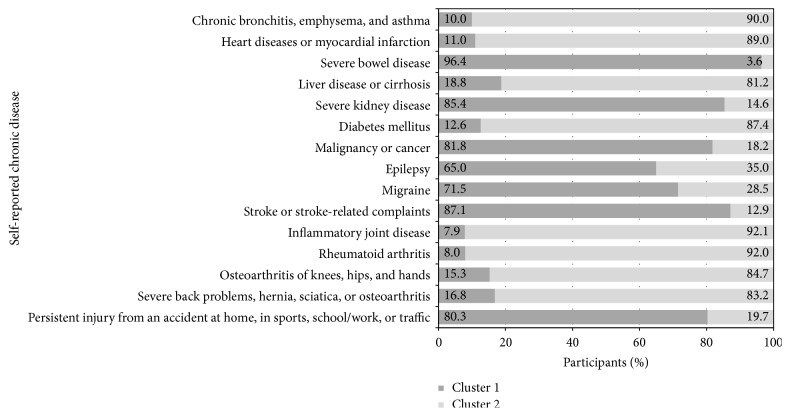
The distribution of patients that suffer from one of the 15 chronic diseases across the two clusters.

Figure 1 shows for each disease how the patients (i.e., the patients that have the disease in question) are distributed across the two clusters. For instance, the first bar in the figure shows that of the patients who have chronic bronchitis, emphysema, and asthma, 10% are assigned to cluster one and 90% are part of cluster two. Detailed information about the importance and distribution of each chronic disease in the clustering can be found in Appendix A.

Of the patients who have severe bowel disease 96.4% are included in cluster one. Of the patients with severe kidney disease or cancer also the majority is involved in cluster one (85.4% and 81.1%, resp.). Similarly of the patients with epilepsy (65.0%), migraine (71.5%), stroke, or stroke-related complaints (87.1%) and persistent injury from an accident at home, in sports, school/work, or traffic (80.3%) the majority is a member of the first cluster. In other words, cluster one is the dominant cluster for severe bowel disease, severe kidney disease, cancer, epilepsy, migraine, stroke, and persistent injury from an accident.

Cluster two is dominated by respiratory disease, heart disease, liver diseases, diabetes mellitus, inflammatory joint disease, rheumatoid arthritis, osteoarthritis, and severe back problems. Of the patients with chronic bronchitis, emphysema and asthma 90.0% are in cluster two. Of the patients with myocardial infarction 89.0% are in cluster two and 81.3% of the patients suffering from liver disease or cirrhosis are included in the second cluster. The majority of the patients with diabetes mellitus (87.4%), inflammatory joint disease (92.1%), rheumatoid arthritis (92.0%), osteoarthritis of knees, hips, and hands (84.7%), and severe back problems, hernia, sciatica, or osteoarthritis (82.3%) are also member of cluster two.

**Figure 2:**
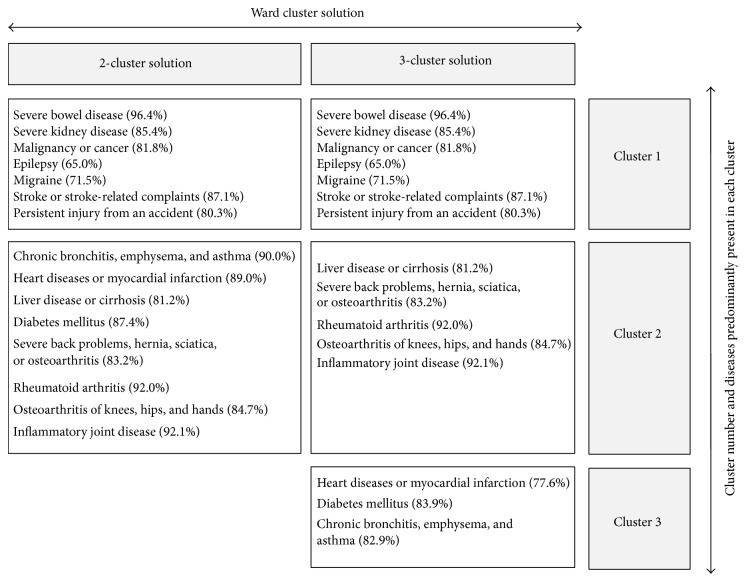
Description of identified clusters according to Ward's agglomerative hierarchical two- and three-cluster solution.

A resumed description of the two clusters is also presented in Figure 2.

#### 3.2.1. Association between Clusters and Physical Activity

*Two-Cluster Solution*. Of the total of 3,386 patients, 60.8% (*N* = 2,060) complied with the Dutch physical activity (PA) guideline. Of the people belonging to cluster one, 61.8% complied with the Dutch PA guideline, and, of the people belonging to cluster two, 59.4% complied with this guideline. The proportion of respondents that complied with the Dutch PA guideline was not significantly different between the two clusters (chi-square: 1.847; *p* = 0.174).

Although statistically a two-cluster solution was identified as being most optimal, the aim of this study was to discover the combination of diseases that not only cluster but also interact with physical activity. To explore whether further branching of clusters might provide information regarding the relationship between clusters and physical activity, analysis proceeded with a Ward's three-cluster solution.

### 3.3. Multimorbidity Clusters

*Three-Cluster Solution*. The results of Ward's three-cluster solution are presented in Figure 2, with Ward's two- and three-cluster solutions shown on the horizontal axis. The boxes below each cluster solution represent the clusters and contain the diseases in each cluster. Ward's three-cluster solution showed that the first cluster remained the same while cluster two separated further (Figure 2). The third cluster contained patients the majority of whom had heart disease or myocardial infarction (77.6%), diabetes mellitus (83.9%), and/or chronic bronchitis, emphysema, and asthma (82.9%).

#### 3.3.1. Association between Clusters and Physical Activity

*Three-Cluster Solution*. The proportion of adults that comply with the Dutch PA guideline is highest in cluster two (62.4%), followed by cluster one (61.8%) and finally cluster three (55.8%). The relationship between the three-disease clusters and PA guideline compliance was statistically significant (chi-square: 7.968; *p* = 0.019).

Ward's four-cluster solution led to a cluster containing a single disease (heart disease). First, because a single disease does not represent a multimorbidity cluster and hence does not fit the aim of the present study, clustering was stopped after Ward's three-cluster solution. Second, all other clusters presented in the four-cluster solution were comparable which supports our decision to stick to the three-cluster solution (Appendix B).

## 4. Discussion

The aim of the present study was to assess the relationship between multimorbidity clusters and compliance with the Dutch physical activity (PA) guideline. The two-cluster solution showed no significant association with PA guideline compliance. Further exploration revealed a significant relationship between three multimorbidity clusters and physical activity. The highest rate of PA guideline compliance (62.4%) was found in cluster two, of which the majority of patients had liver disease, back problems, rheumatoid arthritis, osteoarthritis, and inflammatory joint disease. The lowest rate of PA guideline compliance (55.8%) was reported in patients with heart disease, respiratory disease, and diabetes mellitus. Compared with the average Dutch proportion of older adults (e.g., 68.6% [21, 22]), fewer people adhered to the Dutch physical activity guideline in all three clusters.

The main limitation of the present study is its cross-sectional design, which prevents the establishment of any causal inference. The quantity of missing values ranging from 17.5% (epilepsy) to 24.2% (inflammatory joint disease) in the self-reported chronic disease questionnaire formed a limitation. To obtain as much information as possible, we interpreted missing values as absence of the disease, and this may have caused the disease burden in this population to have been underestimated. As a control, patient characteristics were checked, revealing comparable results for patients with and without missing data on chronic diseases. Furthermore, the presence of chronic diseases was measured via a self-reported questionnaire, and one may well wonder whether a patient is able to report this information adequately. Informed consent issues prevented us from being able to check the self-reported data against data registered in electronic medical records (EMRs). Nevertheless, previous research on the SMILE cohort identified a high level of agreement between self-reports of chronic diseases and information from EMRs [23]. The high level of agreement between medical records and patients' reports in this large community-based cohort supports the accuracy of self-reported data used in answering the research question. The self-reported chronic disease questionnaire could be considered limited and without any assessment of disease severity, and this may have led to an under- or overestimation of the true burden of chronic diseases. Moreover, people tend to overestimate their physical activity level [24], which might have introduced another systematic bias. Also not considered were seasonal influences that could influence the amount of PA performed. Yet, the SQUASH questionnaire represents a reliable and valid measurement instrument for population samples [15]. Finally, while a measure of social desirability may also have influenced the patients' answers, the respondents remained anonymous to researchers and were assured that their information would not be reported to their general practitioner. Despite these limitations, this study is the first to examine the relationship between clusters of chronic diseases and physical activity.

The first analysis revealed two clusters for which no association with PA was detected. The clusters found were broad (representing at least seven diseases) and diverse in terms of types of the diseases embodied in each cluster. As previous research had shown an inverse relationship between multimorbidity and PA, the question of which specific disease combinations are associated with PA remained unanswered. Therefore, the exploration was continued with the three-cluster solution and we found that only the initial second cluster had branched out into two new ones. The results of the three-cluster solution showed that cluster one remained unchanged and that heart disease, respiratory disease, and diabetes had separated from the original cluster two to form a third cluster. The relationship between the three-cluster solution and PA was significant. The third cluster had the lowest proportion of people who were compliant with the Dutch PA guideline. The highest proportion of people who were compliant was found in cluster two, which had a compliance proportion similar to cluster one.

As people in cluster three showed lower activity levels on average, it might be worthwhile to examine the diseases found in this cluster, namely, heart disease, respiratory disease, and diabetes mellitus. It may not be surprising that this combination of diseases formed a separate cluster given that they are highly prevalent diseases that have been shown to be interrelated. For example, Howard et al. [25] estimated that the relative risk of developing cardiovascular disease is two to eight times higher in people with diabetes mellitus compared with nondiabetics. The relationship between respiratory disease and cardiovascular disease seems to be related to systemic inflammation and chronic infections [26]. Systemic inflammation also seems to contribute to the triangle association as there seem to be increased inflammatory markers in diabetes mellitus and respiratory disorders. Reactive Oxygen Species (ROS) injure the airways and promote inflammation and are considered an underlying cause of insulin resistance. Moreover, all three diseases may be intimately intertwined because they share the same risk factors (e.g., smoking, obesity, hyperlipidaemia, and hypertension) [27].

The fact that the diseases in cluster three showed the lowest proportion of PA guideline compliance could be expected. The inverse relationship between cardiovascular disease, respiratory disease and diabetes mellitus, as individual disorders, and physical activity has been studied extensively [25–27].

To our knowledge, only four studies have until now investigated the relationship between multimorbidity and PA [10, 11, 28, 29]. Three of these four studies found an inverse relationship between multimorbidity and physical activity levels [10, 11, 29]. The results of these studies concur with those presented by Hudon et al. [28] who reported that multimorbidity was not associated with physical activity levels. Measurement differences in the assessment of multimorbidity and PA challenge the comparability of results. First, regarding the estimation of chronic diseases, correspondence existed as all four studies used self-reported data and counted the number of chronic diseases. Nevertheless, the chronic diseases listed in the survey or questionnaire and the cut-off point of the disease count defining multimorbidity were dissimilar. Second, differences in PA measurement might have contributed to the variation observed in the results as physical activity is a complex and multidimensional dependent variable which makes population-based measurement difficult. Kaplan et al. [10] asked patients to report the number of times in the past month that they had taken part in recreational PA lasting ≥ 15 minutes. Similarly, Hudon et al. [28] measured PA by the number of recreational PA sessions of 20–30 minutes during the preceding three months. The PASE, an instrument that measures the level of physical activity for individuals aged 65 years and older, was used in the study of Autenrieth et al. [11]. The PASE is comprised of self-reported occupational, household, and leisure items over a one-week period. However, to reach sufficient content validity van Poppel et al. [30] recommended in 2010 (after the study of Kaplan et al. [10] and Hudon et al. [28] had been published, but before Autenrieth and colleagues started their investigation) that each questionnaire assessing total physical activity should at least measure duration and frequency in all settings (household, work, transport, recreation, and sport). Both the International Physical Activity Questionnaire (IPAQ) used by Cimarras-Otal et al. [29] and the Short Questionnaire to Assess Health-Enhancing Physical Activity (SQUASH), which was used in this study, follow this recommendation. The IPAQ and the SQUASH questionnaires allow for a more detailed assessment as they include questions on activity frequency, duration, and intensity and make it possible to determine if a person meets the current recommendation for physical activity. It is important to emphasise that multimorbidity was classified into categories (0, 1, 2, and ≥3 diseases) in all four previously conducted studies. This study is the first which explores the relationship with PA using chronic disease clusters. Investigating the relationship between the number of chronic diseases and compliance with the Dutch PA guideline in the present SMILE cohort study revealed a statistically significant inverse relationship (*p* = 0.004). Although in the present study a cluster analysis was performed because contentwise it fitted our primary aim best, other data reduction methods and procedures are expected to reveal comparable groups of patients [31, 32].

In conclusion, this study adds to our knowledge of the relationship between multimorbidity and physical activity. In addition to the inverse relationship of the number of chronic diseases and PA, the present study showed that the cluster of patients with cardiovascular disease, respiratory disease, and/or diabetes type II reported the lowest physical activity levels. Belonging to a specific cluster of diseases does make a difference and it is important for general practitioners and physiotherapists to help especially patients with cardiovascular disease, respiratory disease, and/or diabetes to initiate and maintain appropriate physical activity levels. It seems worthwhile to further explore the relationship between multimorbidity clusters and outcomes like physical activity, because it helps to deliver more targeted and effective care for patients.

## Figures and Tables

**Figure 3:**
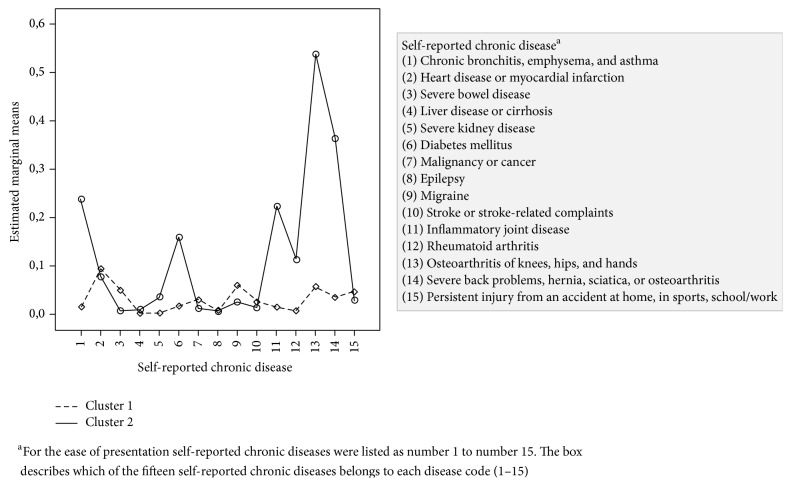
The proportion of patients as a function of chronic disease and cluster division.

**Figure 4:**
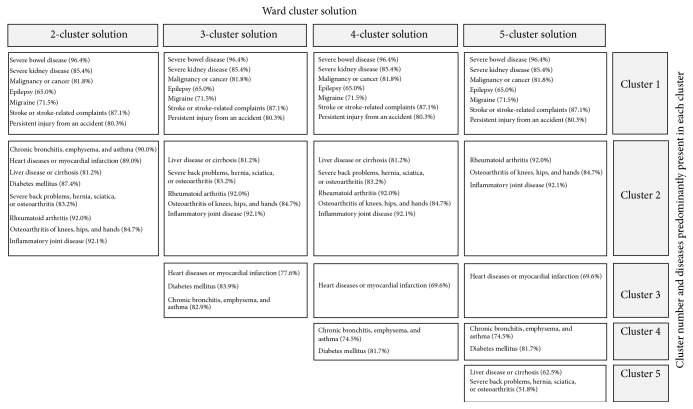
Description of identified clusters according to Ward's agglomerative hierarchical two-to-five-cluster solutions.

**Table 1:** Characteristics of the study population.

Characteristics^a^	Total population *N* = 3,386	Males (47.1%) *n* = 1,595	Females (52.9%) *n* = 1,791
Age (years)	67.5 ± 8.3	67.5 ± 8.2	67.5 ± 8.4
Length (cm)	170.0 ± 8.8	176.2 ± 6.6	164.3 ± 6.5
Body weight (kg)	75.1 ± 13.8	80.4 ± 13.3	70.3 ± 12.3
Chronic bronchitis, emphysema, and asthma	321 (9.5)	148 (9.3)	173 (9.2)
Heart disease or myocardial infarction	299 (8.8)	180 (11.3)	119 (6.6)
Severe bowel disease	112 (3.3)	51 (3.2)	61 (3.4)
Liver disease or cirrhosis	16 (0.5)	9 (0.6)	7 (0.4)
Severe kidney disease	48 (1.4)	25 (1.6)	23 (1.3)
Diabetes mellitus	230 (6.8)	122 (7.6)	108 (6.0)
Malignancy	77 (2.3)	44 (2.8)	33 (1.8)
Epilepsy	20 (0.6)	7 (0.4)	13 (0.7)
Migraine	158 (4.7)	52 (3.3)	106 (5.9)
Stroke or stroke-related complaints	70 (2.1)	35 (2.2)	35 (2.0)
Inflammatory joint disease	302 (8.9)	115 (7.2)	187 (10.4)
Rheumatoid arthritis	150 (4.4)	43 (2.7)	107 (6.0)
Osteoarthritis of knees, hips, or hands	780 (23.0)	290 (18.2)	490 (27.4)
Severe back problems, hernia, sciatica, or osteoarthritis	517 (15.3)	239 (15.0)	278 (15.5)
Persistent injury from an accident at home, in sports, school/work	132 (3.9)	61 (3.8)	71 (4.0)

^a^Dichotomous variables are presented as *N* (%) and continuous variables as the mean ± standard deviation.

**Table 2:** Agglomerative coefficient and pseudo-*F* statistic for hierarchical clustering.

Number of clusters	Agglomeration last step	Coefficient current step	Score change	Pseudo-*F*	*p* value
2	2847.045	2516.781	330.264	1533.167^b^	0.000
3	2516.781	2307.600	209.181^a^	767.332	0.000

^a^Demarcation point → 2 clusters.

^b^Ratio of between-cluster variance to within-cluster variance largest → 2 clusters.

## Appendices

### A. Importance and Distribution of Each Chronic Disease in the Clustering

Figure 3 shows a plot of the cluster centroids with each disease being a cluster variable. These cluster centroids show for each chronic disease (1–15) and for each cluster (dotted and straight line) the proportion of patients (i.e., subjects who have the disease in question). To illustrate, the proportion of subjects with chronic bronchitis, emphysema, and asthma is higher in cluster two compared to cluster one. Moreover, based on these proportions one may identify which chronic diseases are most important in distinguishing between the two clusters of subjects. Clusters one and two differ predominantly with regard to the proportion of occurrence of chronic bronchitis, emphysema, and asthma (21.5% versus 1.6%, resp.); heart diseases or myocardial infarction (1.6% versus 19.8%, resp.); diabetes mellitus (1.4% versus 14.9%, resp.); inflammatory joint disease (1.2% versus 20.7%, resp.); osteoarthritis of knees, hips, and hands (5.8% versus 49.1%, resp.); and severe back problems, hernia, sciatica, or osteoarthritis (4.3% versus 31.9%, resp.).

### B. Identified Clusters according to the Two-to-Five-Cluster Solution

See Figure 4.
